# Seasonal variation in exploitative competition between honeybees and bumblebees

**DOI:** 10.1007/s00442-019-04576-w

**Published:** 2019-12-16

**Authors:** Veronica R. Wignall, Isabella Campbell Harry, Natasha L. Davies, Stephen D. Kenny, Jack K. McMinn, Francis L. W. Ratnieks

**Affiliations:** 1 Laboratory of Apiculture and Social Insects, School of Life Sciences, University of Sussex, Brighton, East Sussex BN1 9QG UK; 2 Department of Zoology, University of Cambridge, Downing Street, Cambridge, CB2 3EJ UK

**Keywords:** Seasonality, Ecology, Competitive exclusion, Bees, Resource availability

## Abstract

Honeybees (*Apis mellifera*) and bumblebees (*Bombus* spp.) often undergo exploitative competition for shared floral resources, which can alter their foraging behaviour and flower choice, even causing competitive exclusion. This may be strongest in summer, when foraging conditions are most challenging for bees, compared to other times of the year. However, the seasonal dynamics of competition between these major pollinator groups are not well understood. Here, we investigate whether the strength of exploitative competition for nectar between honeybees and bumblebees varies seasonally, and whether competitive pressure is greatest in summer months. We carried out experimental bee exclusion trials from May to late September, using experimental patches of lavender, variety Grosso, in full bloom. In each trial, we compared the numbers of honeybees (HB) foraging on patches from which bumblebees had been manually excluded (bumblebee excluded, BBE) versus control (CON) patches, HB_(BBE-CON)_. This measure of exploitative competition varied significantly with season. As expected, mean HB_(BBE-CON)_ was significantly greater in summer trials than in spring or autumn trials. This was despite high nectar standing crop volumes in BBE patch flowers in spring and autumn trials. Mean HB_(BBE-CON)_ was not different between spring and autumn trials. Our results show that nectar competition between honeybees and bumblebees varies seasonally and is stronger in summer than spring or autumn, adding to current understanding of the seasonality of resource demand and competition between bee species. This information may also help to inform conservation programs aiming to increase floral resources for bees by showing when these resources are most needed.

**Electronic supplementary material:**

The online version of this article (10.1007/s00442-019-04576-w) contains supplementary material, which is available to authorized users.

## Introduction

Exploitative competition, in which one consumer species depletes a resource used by other species or individuals (Wootton 1994), can play an important indirect role in shaping community structure and can cause competitive exclusion (Alley 1982; Schoener 1983; Kreutzer and Lampert 1999; Balfour et al. 2015a). More generally, exploitative competition can have a wide range of effects on competing species including behavioural change in resource-use and niche partitioning (Hardin 1960; Inouye 1978; Carpenter 1979; Finke and Snyder 2008; Clink et al. 2017). The strength of competitive pressure for shared resources is expected to vary in response to per-individual resource availability, which can change seasonally in both temperate (e.g. Schmitt and Holbrook 1986; Balfour et al. 2018) and tropical (e.g. Knott 1998; Clink et al. 2017) areas. This can cause species with overlapping foraging niches to seasonally adjust their behaviour (Schmitt and Holbrook 1986), which may mitigate the effects of competition. For example, in seven co-occurring North American waterfowl species, greater competition during resource-scarce winter months caused greater specialisation in food type, habitat utilisation and foraging behaviour between species pairs compared to summer (DuBowy 1988). Conversely, seasonal periods of resource abundance can cause shifts in behavioural strategies through reduced intra- and inter-specific exploitative competition. For example, primates exhibit greater dietary selectivity when fruit availability is high in ‘mast’ years (Knott 1998; Clink et al. 2017).

Seasonal variation in exploitative competition among bee species would be expected to influence foraging behaviour, since many species are generalist nectar-feeders that can flexibly alter the flower species they visit in response to resource availability. Flower choice can be influenced directly by changes in reward quality or quantity (Heithaus 1979; Cnaani et al. 2006) or indirectly through fluctuations in competitor abundance (Heinrich 1976; Walther-Hellwig et al. 2006; Fontaine et al. 2008; Balfour et al. 2015a). Several studies have shown exploitative competition between bee species (e.g. Heinrich 1976; Inouye 1978; Ings et al. 2006, Walther-Hellwig et al. 2006; Balfour et al. 2015a), but these have largely been carried out at a particular time of year and so do not address possible seasonal variation. An April to September study of four heathland sites in southern England provided some evidence of seasonal change in the foraging-niche breadth of long-tongued bumblebees with increasing honeybee abundance, but it was not clear whether this was due to competition (Forup and Memmott 2005). Nevertheless, it is probable that the strength of exploitative competition for nectar among bee species does vary seasonally in many locations. Waggle dance decoding showed that honeybee foraging distances were greatest during July and August in Sussex, southeast England, suggesting a relative scarcity of available floral resources at this time of year compared to spring and autumn (Couvillon et al. 2014a). Since many bee and other flower-visiting insect species also demonstrate a July–August summer peak in abundance in the UK (Falk 2015; Balfour et al. 2018), it is likely that these factors combine to cause a predictable, seasonal, summer increase in nectar competition.

Honeybees (*Apis mellifera*) and bumblebees (*Bombus* spp.) are generalist bees that overlap in floral resource use (Steffan-Dewenter and Tscharntke 2000; Forup and Memmott 2005; Thomson 2006) and are known to undergo inter-specific resource competition, which can affect foraging patterns and behaviour in both groups (e.g. honeybees, Balfour et al. 2015a, b; bumblebees, Sáez et al. 2017). *Apis*-*Bombus* resource competition has also been shown to cause fitness costs (reduced growth and reproduction) in bumblebees (Thomson 2004; Goulson and Sparrow 2009; Elbgami et al. 2014) though not honeybees in the existing literature (reviewed in Wojcik et al. 2018).

Both *Apis* and *Bombus* often occur in large numbers on flowers relative to other bees and insects (Garbuzov and Ratnieks 2014b) due in part to their large eusocial colonies (Seeley 1995; Goulson 2003). The absolute and relative abundance of *Apis* and *Bombus* changes over the foraging season in the UK. Bumblebees have annual colonies and are less abundant in spring/early summer and autumn when colonies are in the stages of growth and senescence, respectively (Falk 2015). In comparison, honeybees have perennial colonies and undergo much smaller seasonal fluctuations in numbers, with foragers active from March to October in our study area (Garbuzov and Ratnieks 2014a; Couvillon et al. 2014a) and often even earlier and later in the year. Therefore, seasonal changes in both competitor abundance and resource availability could cause seasonal change in the strength of *Apis*-*Bombus* exploitative competition. However, our knowledge of this is currently limited despite the increasing (see Breeze et al. 2011) importance of these bees for the pollination of crop and wildflower plant species (Corbet et al. 1991; Carreck and Williams 1998; Woodcock et al. 2013; Garratt et al. 2014), and the potential effects of *Apis*-*Bombus* floral resource competition on bee fitness (growth and reproduction; Thomson 2004; Goulson and Sparrow 2009), foraging behaviour (Walther-Hellwig et al. 2006; Nielsen et al. 2017) and pollination effectiveness (Greenleaf and Kremen 2006).

Previous research in July and August has shown that bumblebees displace honeybees via exploitative competition on patches of lavender flowers (*Lavandula* x *intermedia* ‘Grosso’). Bumblebees outcompete honeybees in this system because they are able to visit Grosso lavender flowers at three times the rate of honeybees (Balfour et al. 2013), which depletes nectar levels to a point at which honeybees cannot make an energy profit. When bumblebees were experimentally excluded honeybee numbers increased 14-fold in response to reduced resource depletion, demonstrating ecological release from competition (Balfour et al. 2015a).

In this project, we aim to determine the seasonal dynamics of *Apis*-*Bombus* exploitative competition on lavender flowers. We extend the previous research to incorporate seasonality by carrying out foraging exclusion experiments from late May to September 2017 on patches of Grosso lavender in full bloom, thereby extending the period over which *Apis*-*Bombus* competition is studied. Importantly, we use a single plant variety thereby controlling the resource. We test the hypotheses that the strength of *Apis*-*Bombus* competition for nectar i) varies over a foraging season and ii) is greater in summer than in spring and autumn.

## Methods

### Study site and species

Field work was carried out on the University of Sussex campus in southeast England (50.8671° N; 0.0879° W). We repeated 10 identical three-day exclusion trials from May to September 2017. Data on bee foraging were collected only on days considered suitable for foraging, > 12 °C, with light winds and no rain, when honeybees and bumblebees were seen to be actively foraging on the lavender plants and/or on other flowers in the study area. There were two apiaries belonging to the Laboratory of Apiculture and Social Insects within < 1 km of the study site each (with between 6 and 10 colonies in total during the study period), plus three further apiaries within < 2 km, and a high density of colonies managed by beekeepers in the wider local area. Honeybees mainly forage for nectar and pollen from March to October (Couvillon et al. 2014a), and healthy colonies consist of between 20 and 40,000 adult bees in May–June and some 40,000 in September (Hooper 1991). Therefore, it is certain that foraging honeybees were present and abundant in the area throughout the study period.

We used the same lavender variety, *Lavandula* x *intermedia* ‘Grosso’ (Lamiaceae), as the previous research that demonstrated exploitative competition for nectar between *Apis* and *Bombus* in summer (Balfour et al. 2013, 2015a).

A total of 700 Grosso plants in 3 L pots were obtained from Downderry Nursery, Sussex (www.downderry-nursery.co.uk), the same supplier as for the previous competition studies (Balfour et al. 2013, 2015a). The plants had been grown in ways to cause bloom at different times. 300 plants were kept in greenhouses and polytunnels by Downderry Nursery to induce early flowering in May and June. 150 plants were grown normally, without treatment, and flowered in late July. A final batch of 250 plants were trimmed during the summer to delay bloom until September, with 150 plants used in the final two trials, 9 and 10. Some of this batch flowered in late August and 96 spare plants were used to replace plants that were near the end of their bloom in Trial 8 (21–24 August), to ensure a similar level of bloom across trials. Different growth regimes did not affect the general appearance of the plants and average nectar secretion rate was similar between batches (Results).

### Trial design and experimental exclusions

The May to September study period was categorised into three seasons, spring (May and June), summer (July and August), and autumn (September). July and August were combined as summer since honeybee foraging distances are highest in the study area in these months, which indicates a dearth in overall nectar availability (Couvillon et al. 2014a). Pre-July study months were combined as spring. Autumn was defined according to the National Met Office definition of meteorological autumn as starting on 01 September (National Met Office 2019), and also coincided with the flowering of ivy (*Hedera* spp.) in the study area from early September, following Couvillon et al. (2014a).

Each trial consisted of three exclusion days. Exact trial dates were dependent on suitable weather conditions. We aimed to carry out an even number of trials per season, but this was not possible due to poor weather conditions in spring and the lack of lavender plants in full bloom in autumn following the final trial. We achieved three trials in spring (1–3: 23–25 May, 31 May–02 June and 13–15 June), five in summer (4–8: 04–06 July, 10–13 July, 31 July–04 August [data not collected on 02–03 August due to bad weather], 14–16 August and 21–23 August) and two in autumn (9–10: 12–14 and 19–22 September) making ten in total. We alternated trials between two sites 600 m apart on the University campus to reduce any potential local-effect bias.

Following Balfour et al. (2015a), each three-day trial was set up using 150 plants in three patches of 50 pots, separated by 100–200 m. Plants were selected at the start of the trial to give approximately equal total bloom per patch. Each patch was randomly assigned to a treatment: honeybees excluded (HBE), bumblebees excluded (BBE) and control (CON, no bees excluded). Following established methods (Balfour et al. 2015a), bees of the “wrong” type were excluded throughout each day using a light tap with a bamboo cane. On all patches we excluded male wool carder bees (*Anthidium manicatum*), since these are highly territorial and aggressive towards other bee species, and the conopid fly (*Sicus ferrugineus)* which lays its eggs on foraging bumblebees (Falk 2015), in case these insects were causing honeybees and bumblebees to avoid the lavender; both were rarely present.

We estimated the total number of flowers in each patch once during each trial by counting the number of flowering inflorescences in the patch and multiplying this by the average number of flowers calculated from 40 randomly-selected inflorescences.

### Bee count data

Data collection followed established and effective methods for counting bees visiting flowers (Garbuzov and Ratnieks 2014b; Balfour et al. 2015a). We counted bees foraging in each patch from 09:00 to 17:30 on each trial day. To do this we made a near instantaneous count every 30 min in which we scanned the patch by eye for approximately 30 s and recorded any bees and other insects actively foraging at that time (Garbuzov and Ratnieks 2014b). In general, bees spend < 30 min in a patch during a single foraging attempt. Therefore, although individuals will revisit patches, the 30-min interval between counts means that the count data represent different visits (Garbuzov and Ratnieks 2014b). After 17:30 all patches were covered with netting to prevent insect access until targeted exclusions resumed the following morning (Balfour et al. 2015a).

Bumblebees, including parasitic cuckoo species (subgenus *Psithyrus*), were mostly identified according to species. The two-banded white-tailed bumblebees *Bombus terrestris* and *B. lucorum* are difficult to distinguish in the field and were grouped as *B. terrestris/lucorum* (Fussell and Corbet 1992). Solitary bees were identified according to species where possible, or to genus. Any bees that could not be recognised by eye were caught and identified using a hand lens or microscope. The vast majority of foragers were collecting nectar only and were only ever observed carrying trace amounts of pollen, supporting previous observations in which less than 5% of the foragers on Grosso were observed with pollen in their corbiculae (Balfour et al. 2013).

### Nectar measurement

During each trial we measured secretion rate, standing crop and sugar concentration using microcapillary pipette tubes (Drummond Microcaps 1 µL, 64 mm, 1-000-0010-64 or 0.25 µL, 32 mm, 1-000-00025) inserted into an open flower to extract the nectar from the base of the corolla. The length of nectar drawn up into the tube was measured using a ruler and used to calculate the per-flower volume of nectar as a proportion of the overall tube volume (Corbet 2003; Balfour et al. 2013). Each microcap was used a single time only (Corbet 2003). Nectar measurements were made once per trial, between 12:00 and 14:00 to minimise day-to-day variation.

To measure the per-flower volume of nectar available to insects (standing crop) we extracted nectar from 10 flowers in each patch. Nectar sugar concentration (% Brix) was measured for each sample with sufficient volume using a hand-held refractometer (Bellingham and Stanley^TM^, 0–50% Brix). To measure hourly nectar secretion rate per flower we used microcaps to empty as fully as possible several flowers in the CON patch, taking care not to damage the nectaries (Corbet 2003). We marked these flowers and bagged the entire inflorescence using fine gauze bags to prevent insect access. After 60 min, we extracted nectar from the marked flowers individually and recorded the volume of liquid contained in the microcap.

### Statistical analysis

We analysed seasonal changes in honeybee visits to lavender flowers when bumblebees were manually excluded (BBE patch) relative to the control (CON) patch over ten trials. The following statistical analysis uses the second and third exclusion days of each trial, when bee numbers and foraging behaviour had stabilised following one full day of exclusions. This is because we observed that honeybee numbers on the BBE patch often varied considerably over the course of the first trial day, which is consistent with previous research in which honeybee numbers took approximately 1.5 days to plateau following the start of bumblebee exclusion from lavender patches (Balfour et al. 2015a, b). To remove this noise in the data, we removed the first trial days from analysis.

As a proxy measure of competition we calculated the absolute difference in per-day mean honeybee counts from 09:00 to 17:30 (*n* = 18 counts per day) between the two patches [(mean HB_(BBE)_)–(mean HB_(CON)_)], hereafter HB_(BBE-CON)_, since this metric gives a clear indication of the increase in honeybee visits to the BBE patch compared to the control. Using daily average counts removed pseudo-replication from the raw data, and normalised the positively skewed distribution, thereby also correcting for overdispersion. HB_(BBE-CON)_ also accounts for any between-trial variation in the number of flowers.

To analyse between-season variation in HB_(BBE-CON)_ we used a linear mixed effects model [lmer, package lme4 (Bates et al. 2015)] with per-day HB_(BBE-CON)_ as the response (*n* = 20) and season (spring, summer, autumn) as a fixed effect. Trial was included in the model as a random effect since we expected between-trial variation in HB_(BBE-CON)_, but were not directly testing differences in the response between specific trials in this model (Bolker et al. 2009). Trial day (2 or 3) and site were added as interaction terms to assess any confounding effect on HB_(BBE-CON)_ with the effect of season, but neither were significant and so were not included in the final model. Residuals were visually checked for normality and homoscedasticity, and approved. Differences between seasons were calculated using post hoc pairwise comparisons across groups, using lsm [package lsmeans (Lenth 2016)] within glht [package multcomp (Hothorn et al. 2008)], with *P* values adjusted for multiple comparisons by the single-step method.

**Fig. 1:**
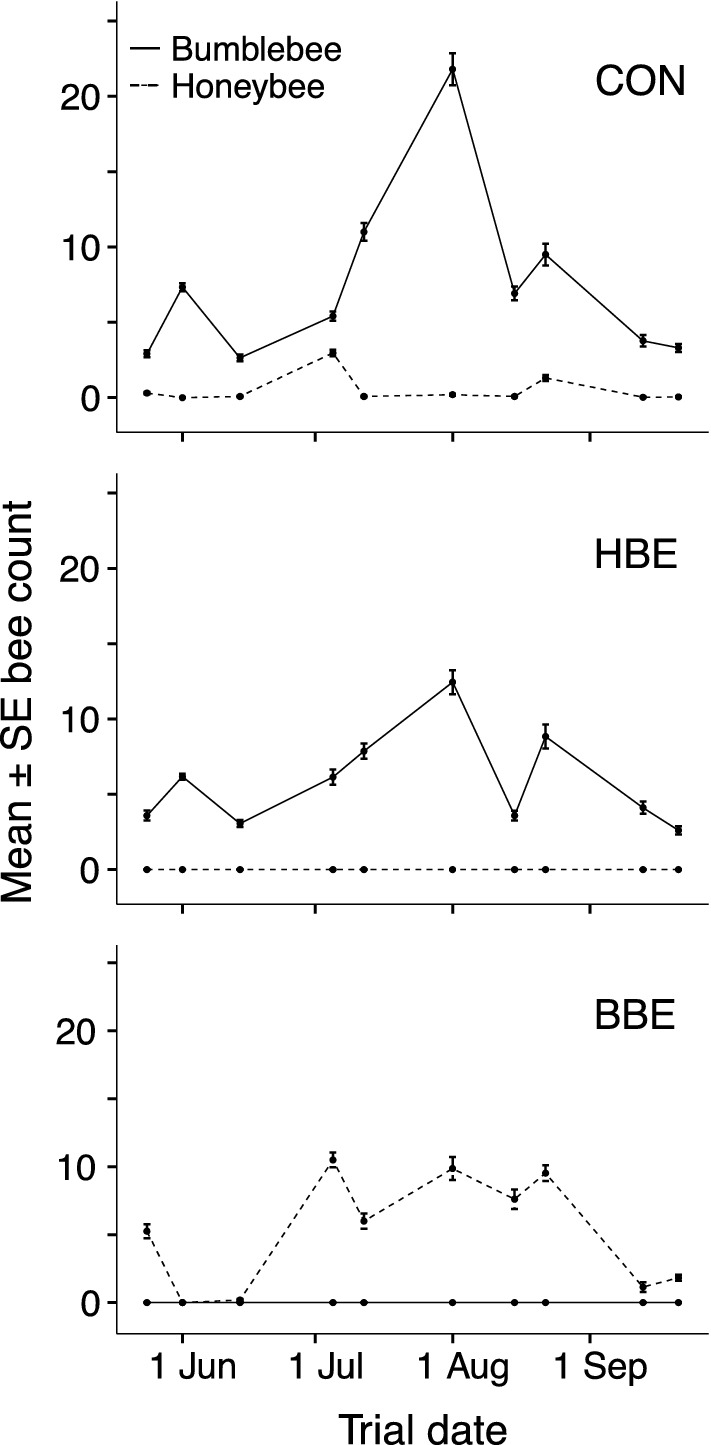
Numbers of honeybees (*Apis mellifera,* dashed lines) and bumblebees (*Bombus* spp., solid lines) foraging on lavender patches from which bumblebees have been excluded (BBE), honeybees have been excluded (HBE), and unmanipulated control patches (CON), across ten trials from May to September 2017. Points show the mean count per day averaged over trial days 2 and 3 (*n* = 36 = 2 days × 18 counts per day from 09:00–17:30). Error bars show ± 1 SE

We did not expect honeybee exclusion (HBE) to impact bumblebee visitation, given previous results (Balfour et al. 2015a), although a seasonal effect was possible and worth investigating since the previous study was conducted only in summer (July–August). In fact, honeybee abundance on control patches was consistently low, and there was no increase in bumblebee numbers on the HBE patch relative to the control (Fig. 1; Online Resource 1). Therefore, seasonal changes in bumblebee abundance were not explored statistically. We also did not analyse the effects of honey- and bumblebee exclusion on other insect groups since frequencies of these were too low for statistical analysis.

Nectar standing crop volumes were compared between patch treatments in each trial using per-trial Kruskal–Wallis H tests and post hoc Dunn’s tests for pairwise comparisons between treatments with Bonferroni adjustment of *P* values (results in Online Resource 3). One-hour nectar secretion rates were compared between batches of lavender plants (*n* = 4 batches) and between trials (data available for eight of 10 trials) using Kruskal–Wallis *H* tests for non-parametric data.

Significance was defined at *P* < 0.05. All analyses were performed using R Studio Version 1.1.419.

## Results

### Insect abundances on the control patch

Almost all insects (96.4%) observed foraging on the lavender control (CON) patches over the 10 trials were bumblebees (90.4%) and honeybees (6.0%). Other foraging insects included butterflies and moths (1.7%), hoverflies (0.7%) and solitary bees (0.6%). The remaining 0.6% were classified as other insects and were mainly non-Syrphidae Diptera. The number of honeybees per count on the control patches was consistently low, often 0, with bumblebees approximately 15 times more numerous (overall mean ± SD: 0.51 ± 1.09 honeybees v. 7.46 ± 6.30 bumblebees, n = 10 trials; Fig. 1). The abundance and species composition of bumblebee foragers on the control patch were variable over the study period, with *Bombus terrestris/lucorum* and *B. pascuorum* most frequent (Online Resource 4).

### Honeybee response to bumblebee exclusion

**Fig. 2:**
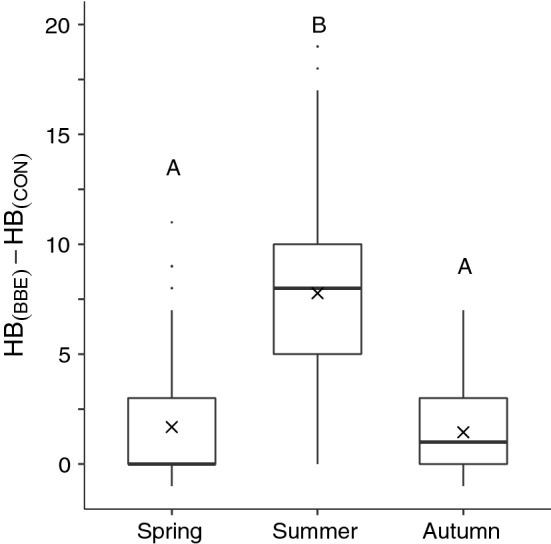
Seasonal change in HB_(BBE)_–HB_(CON)_ between spring (*n* = 3 trials), summer (*n* = 5 trials) and autumn (*n* = 2 trials) 2017. HB_(BBE)_–HB_(CON)_ signifies the number of honeybees foraging on lavender plots from which bumblebees had been excluded (BBE) compared to unmanipulated control patches (CON; *n* = 18 counts per day from 09:00–17:30). All data are from trial days 2 and 3. Boxplot limits are the 25th and 75th percentiles, whiskers are 1.5 × the interquartile range, horizontal lines indicate the median, crosses (×) within plots represent the mean (described as HB_(BBE-CON)_ in the text) and points outside whiskers represent outliers. Initials above plots (A, B) denote significance between per-season HB_(BBE-CON)_ means, defined at *P *< 0.05

The per-trial mean number of honeybees foraging on the bumblebee excluded (BBE) patch compared to the control patch (HB_(BBE-CON)_) varied significantly according to season (LMER: $\chi_{(2)}^{2}$ = 28.5, *P *< 0.001, *n* = 36 counts per trial; Fig. 2). Importantly, the effect of bumblebee exclusion, mean HB_(BBE-CON)_, per trial, was substantially and significantly greater in summer trials (mean ± SD, 7.77 ± 4.02) than in spring (1.69 ± 2.9, GLHT:LSM post hoc*, t*_(7)_ = 4.55, *P *= 0.0063) or autumn trials (0.68 ± 2.03, GLHT:LSM post hoc, *t*_(7)_ = 4.13, *P *= 0.0108). Mean HB_(BBE-CON)_ was not significantly different between spring and autumn trials (GLHT:LSM post hoc, *t*_(7)_ = 0.14, *P *= 0.99).

**Fig. 3:**
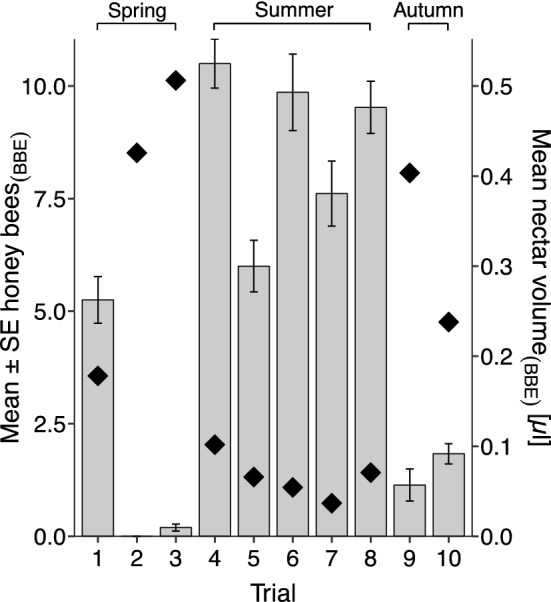
Mean number of foraging honeybees and nectar standing crop volume per flower on lavender patches from which bumblebees have been excluded (BBE) across ten trials from May to September 2017. Bars show the mean per-trial honeybee count (*n* = 18 counts per day from 09:00–17:30), error bars show ± 1 SE. Filled diamonds indicate mean nectar standing crop volume per flower per trial (µL; *n* = 10 flowers per day). Nectar and bee count data for each trial are from days 2 and 3, except Trial 1 in which nectar was extracted only on day 2. Seasons are indicated above the bars: spring (May–June, Trials 1–3); summer (July–August, Trials 4–8) and autumn (September, Trials 9–10)

In summer the number of honeybees visiting the BBE patch was consistently high in all five trials (mean ± SD, 8.7 ± 4.27 honeybees; Figs. 1, 3). Mean per-trial HB_(BBE-CON)_ ranged from 5.92 ± 3.45 (Trial 5) to 9.67 ± 5.09 (Trial 6) in this season. In autumn, the number of honeybees visiting the BBE patch was consistently low (1.49 ± 1.82 honeybees; Figs. 1, 3), despite many honeybees observed foraging on ivy flowers in close proximity to the study patches. Mean per-trial HB_(BBE-CON)_ was also low, from 1.11 ± 2.17 (Trial 9) to 1.78 ± 1.31 (Trial 10).

In spring there was clear variation in the number of honeybees foraging on the BBE patch between trials (Figs. 1, 3). In Trial 1, many honeybees were observed foraging on the BBE patch (5.25 ± 3.11 honeybees) compared to zero (0.00 ± 0.00) or few (0.194 ± 0.467) in Trials 2 and 3, respectively. During both Trial 2 and 3, honeybees were seen foraging on bramble flowers and other species of flowering plant in the study vicinity.

### Bumblebee response to honeybee exclusion

There was no increase in bumblebee numbers in response to honeybee exclusion, with similar visitation to HBE and CON patches in each trial (Fig. 1; Online Resource 1). Honeybee numbers on CON patches were low throughout the study period (Fig. 1), and very few were ever excluded from the HBE patches meaning that any effect on bumblebee numbers was likely to be negligible. Therefore, this result is not discussed further.

### Lavender nectar

Overall, mean hourly nectar secretion rate per flower was 0.038 ± 0.002 µL/h^−1^ (mean ± SD, *n* = 154 flowers). Mean hourly secretion rate was not different between four lavender batches grown under different regimes (Kruskal–Wallis *H* test: $\chi_{(3)}^{2}$ = 6.77, *P *= 0.079, *n* = 4 batches), but differed significantly between trials (Kruskal–Wallis H test: $\chi_{(7)}^{2}$ = 25.47, *P *< 0.001, *n* = 8 trials).

Per-trial standing crop of nectar in BBE patch lavender flowers was inversely related to honeybee visitation rates to this patch (see Online Resource 2). When honeybees were visiting the flowers in large numbers, nectar standing crop volumes (both per bee per patch and per bee per 100 flowers) were small, compared to high volumes when bees were visiting in low numbers (Fig. 3).

The following data all refer to the per-trial average nectar standing crop volume extracted from 10 flowers on days 2 and 3 of each trial (*n* = 20 flowers) except Trial 1 in which nectar was extracted only on day 2 (*n* = 10 flowers). Nectar standing crop volume was always low in the CON patch flowers (mean ± SD over ten trials = 0.042 ± 0.078 µL) and in HBE patch flowers (0.043 ± 0.090 µL). Nectar standing crop in the BBE patch (0.210 ± 0.273 µL) was higher than the control patch in every trial, which was significant in all trials apart from 1 and 4 according to per-trial Kruskal–Wallis *H* and post hoc Dunn’s tests for pairwise comparison between patch treatments (Online Resource 3). When averaged within seasons, nectar standing crop volume extracted from BBE patch flowers was 412% higher than the control patch in spring (BBE 0.408 ± 0.276 µL; CON 0.099 ± 0.120 µL), 275% higher in summer (BBE 0.066 ± 0.085 µL; CON 0.024 ± 0.047 µL) and 1783% higher in autumn trials (BBE 0.321 ± 0.361 µL; CON 0.018 ± 0.019).

Nectar standing crop volume in flowers in the HBE compared to CON patch was not significantly different in any trial, according to per-trial Kruskal–Wallis *H* and post hoc Dunn’s tests for pairwise comparison between patch treatments (Online Resource 3).

## Discussion

Our results indicate, for the first time to our knowledge, that the strength of exploitative competition for nectar between two major pollinator groups can vary seasonally. As expected, *Apis*-*Bombus* competition on lavender Grosso flowers was greater in summer than spring or autumn (Fig. 2). On average, in summer trials (July and August) there were 8.70 honeybees foraging on the bumblebees excluded (BBE) patch per count compared to 0.93 honeybees on the control (CON) patch, a near tenfold increase, demonstrating ecological release from competition. In contrast, in spring (May and June) and autumn (September) trials, honeybees were absent or very infrequent on the BBE patch, despite high levels of nectar in the flowers, suggesting the reverse, that competition for nectar was reduced in these periods. This seasonal trend was statistically significant using the metric HB_(BBE-CON)_ to compare the number of foraging honeybees on BBE vs CON patches between seasons.

Our results suggest that competition for nectar was high throughout the summer period. Honeybees consistently visited the bumblebee excluded (BBE) patch in large numbers in each of the five July and August trials, while numbers on the control patch remained low (Fig. 1). Exploitative competition between coexisting species and individuals is expected to be strong when shared resources are limited, as a result of the interaction between the availability of food resources in the landscape and the abundance of competitors. Waggle dance decoding has shown that honeybees forage furthest from the nest in July and August (Couvillon et al. 2014a), and August is also the time with the largest proportion of returning foragers having empty crops (Couvillon et al. 2014b). Since worker honeybees are efficient foragers that rapidly recruit nestmates to exploit the most profitable floral resources (Núñez 1982; Schmid-Hempel 1987; Requier et al. 2015), these studies imply that summer is a period of limited overall nectar availability for bees.

Absolute nectar provision in kilograms per hectare is in fact estimated to be highest in July and August in the UK overall (Baude et al. 2016). However, this is likely to be subject to local effects. For example, summer-flowering heather species *Erica cinerea* and *Calluna vulgaris* together are estimated to have contributed 16.5% of annual national nectar provision in 2007 (Baude et al. 2016), but these are virtually absent in our study area. Additionally, non-woody flowering plants (herbs) make up the majority of insect-pollinated plant species flowering in July and August (Balfour et al. 2018). However, this floral group is known to have suffered extensive declines in the 20th century (Stroh et al. 2014) including significant decreases in the range and frequency of important summer-flowering pollinator forage plants (Carvell et al. 2006). Even if absolute nectar provision is greater in summer, per-insect nectar availability could still be lower in this season if there are many more nectar-feeding insects. A recent study of British phenological records showed that 62% of flower-visiting insect species (71% of aculeate wasp, 60% bee, 72% butterfly and 49% of hoverfly species) peak in abundance in July and August (Balfour et al. 2018). It is, therefore, possible that increased insect abundance and reduced flower availability combine to create a summer increase in competitive pressure for pollinating insects due to lower per-insect nectar availability.

Stronger nectar competition in summer is likely to affect competition between honey- and bumblebees since they are floral generalists that often have a high level of interspecific dietary overlap, particularly for nectar (e.g. Forup and Memmott 2005; Thomson 2006; but see Leonhardt and Blüthgen (2012) for differences in pollen foraging). For example, in a summer foraging ‘hotspot’ for honeybees 2–3 km from our study site, which was identified by waggle dance decoding (Couvillon et al. 2014a), honeybees and bumblebees visited similar flowers in July and August (Balfour et al. 2015b). Additionally, both *Apis* and *Bombus* are eusocial and have substantial colony requirements: a typical honeybee colony requires 20 kg pollen and 120 kg nectar per year (Seeley 1995), while in one study *Bombus terrestris* colonies consumed on average 176 g pollen and 935 g sugar over a 12-week lifecycle (Rotheray et al. 2017). Honeybees and 22 of 27 UK bumblebee species have a summer peak in abundance (Falk 2015; Balfour et al. 2018). Increased demand for limited per-insect nectar and pollen resources in summer is a likely explanation for our findings and previous work showing strong competition between honeybees and bumblebees at this time of the year in the UK (Goulson and Sparrow 2009; Elbgami et al. 2014; Balfour et al. 2015a) and Europe (Walther-Hellwig et al. 2006).

In contrast to summer, in spring and autumn trials we observed that although honeybees were seen visiting flowering plant species in the close vicinity, they foraged infrequently or not at all on the BBE lavender patches despite a much greater nectar standing crop volume in the flowers, on average sixfold greater in spring and fivefold greater in autumn compared to summer (Fig. 3). This strongly suggests that nectar competition was reduced in these seasons, since exclusion of bumblebees caused little or no increase in honeybees: ecological release from competitive displacement was not apparent. It suggests that honeybees did not ‘need’ the lavender nectar in autumn and spring, perhaps due to higher per-insect nectar availability in the wider local environment. This may relate partly to the seasonal bloom of certain wildflowers, which is known to have an ecologically significant impact on the amount of nectar available to bees (Seeley 1995). In autumn the apparent drop in *Apis*-*Bombus* competition was likely due to the blooming of ivy (*Hedera helix*), which is abundant and a major source of pollen and nectar in autumn for many insects (Garbuzov and Ratnieks 2014a; Jacobs et al. 2010). Since ivy significantly impacts foraging behaviour when it is in flower and is likely to cause a marked increase in nectar availability (Couvillon et al. 2014a), its flowering period may also cause a seasonal reduction in inter- and intra-specific exploitative competition between insects foraging at this time of year. Similarly, in spring trials, lower *Apis*-*Bombus* competition overall may have been due to a generally richer floral community in May and June than summer months (Balfour et al. 2018).

Why did honeybees not forage on lavender flowers in spring and autumn trials, despite the absence of the dominant competitor and resultant high nectar standing crop; what mechanism could be involved? A nectar volume of 0.019 µL and 39% sugar concentration resulted in a substantial energetic profit for honeybees foraging on Grosso lavender (Balfour et al. 2015a), enough to cause a 14-fold increase in honeybee numbers. In this study, nectar volume reached a much greater maximum per-trial average of 0.506 µL in BBE patch flowers in spring (concentration 41.4% sugar, *n* = 17 flowers; Trial 3) and 0.404 µL in autumn (concentration 32.8% sugar, *n* = 17 flowers; Trial 9), suggesting that honeybees would certainly have been able to make a significant profit from foraging on the flowers in these seasons.

Although the high nectar standing crop in BBE patch lavender flowers in spring and autumn trials implies that foraging honeybees could make a profit, it is possible that lavender Grosso was nevertheless suboptimal compared to other floral resources in the environment. More abundant nectar availability in these seasons may have reduced recruitment of nestmates to the BBE patch (Seeley 1995). When colony nectar intake is high, honeybee nectar foragers adaptively raise their dance thresholds, meaning that only high-quality food sources are advertised by returning foragers (Seeley 1995). In contrast, in resource-scarce summer months greater honeybee recruitment to the BBE flowers may be explained by a lower colony dance threshold.

Both honeybees and bumblebees are often numerically dominant foragers on a wide range of flower species (N. J. Balfour, unpublished data). This is likely often to impact the foraging behaviour of other common flower-visiting insects including solitary bees, butterflies and hoverflies. In this study, we did not analyse the effects of competitor removal on other insect groups, since these were too infrequent on the lavender flowers for the necessary statistical power. Plants with a greater number of non-*Apis*/*Bombus* insect foragers may be more suitable for experiments in which the exclusion method used here could begin to examine competition between honeybees, bumblebees and other insect taxa through the removal of both *Apis* and *Bombus*, as well as each group separately; this deserves further investigation.

The effect of seasonal fluctuations in exploitative competition between *Apis* and *Bombus* at a population level in areas where both are native is not clear. However, in one UK study conducted in August, workers of four bumblebee species had smaller average thorax size in sites where honeybees were present compared to where they were absent (Goulson and Sparrow 2009). It is possible that there may be negative fitness implications in times of increased competitive pressure, at least for bumblebees, although further research is needed to clarify this. Future research could also investigate whether these possible population-level effects could be compensated for by seasons in which exploitative competition is weaker.

We show here that the strength of competition for a standardised floral nectar resource between bumblebees and honeybees varies seasonally, with a summer peak in July and August. This is similar to previous work in which waggle dance decoding showed that honeybees forage furthest from the hive in July and August, indicating a dearth in environmental nectar availability relative to other times of the year (Couvillon et al. 2014a). Our results, therefore, also help confirm that waggle dance decoding can provide useful information about foraging conditions for honeybees. Honeybee foraging distances are thought to act as an indicator of seasonal foraging challenge for other flower-visiting insects (Couvillon et al. 2014a). We suggest that seasonal trends in competition between honeybees and bumblebees may similarly predict patterns of competitive pressure for floral resources between flower-visiting insects more broadly. While we have studied lavender as a useful phytometer with which to observe changes in *Apis*-*Bombus* competition, future studies should also extend this to include other locations and plant species, including native and wild-growing flowers if possible, to confirm our findings.

Understanding the seasonality of resource demand and competition between bee and other insect species is also important for informed conservation practice (Williams et al. 2015). Many insect species are in decline in Europe and globally (e.g. Potts et al. 2010; Hallmann et al. 2017) and for flower-visitors a major driver is thought to be a widespread loss of floral resources (Goulson et al. 2008; Potts et al. 2010). A need to help insect pollinators may be particularly important in July and August months, when competition for nectar seems to be increased in the UK (Couvillon et al. 2014a; Balfour et al. 2018; this study). Seasonal plant–pollinator interactions are also likely to be affected by climate change, which can be mitigated by increasing floral availability at certain times of the year (Memmott et al. 2010). Overall, there is a clear need to ensure that floral resources for bees and other insects are sustained throughout the foraging season by considering per-insect floral resource availability in local and landscape-scale resource management. A better understanding of seasonal variation in nectar competition can help in achieving this.

## Electronic supplementary material

Below is the link to the electronic supplementary material.

Supplementary material 1 (PDF 66 kb)

Supplementary material 2 (PDF 60 kb)

Supplementary material 3 (PDF 98 kb)

Supplementary material 4 (PDF 227 kb)
