# Supplementary material for: Seasonal variation in exploitative competition between honeybees and bumblebees
Source: Oecologia. 2019 Dec 16;192(2):351–61. doi: 10.1007/s00442-019-04576-w (PMC7002462; doi:10.1007/s00442-019-04576-w)
Supplement: Supplementary file 1 — Supplementary material 1 (PDF 66 kb) [file 442_2019_4576_MOESM1_ESM.pdf]

Online Resource 1

Bumble bee response to honey bee exclusion: Results

On average, there were more bumble bees per count on the control patch in late summer trials (mean ± SD, 10.90 ± 7.05 bumble bees) than in early summer (4.30 ± 2.60) or autumn (3.54 ± 1.96), with changes in species composition from May to September (Supporting Information Fig. S1). However, there was no increase in bumble bee numbers in response to honey bee exclusion, with similar visitation to HBE and CON patches in each trial. In fact, bumble bee abundance on the control patch was higher than on the HBE patch in three of the five late summer trials (Fig. 1). The average difference in daily mean bumble bee abundance on the HBE patch compared to the control ($BB_{(HBE\text{-}CON)}$) was low over all ten trials (mean -1.62 ± 4.19; range -9.36 ± 5.71 (Trial 6) to 0.72 ± 2.51 (Trial 4). After removing Trial 6, in which there were many more bumble bees on the CON patch compared to the HBE patch, the average difference between HBE and CON patches was even smaller ($BB_{(HBE\text{-}CON)}$ = -0.76 ± 2.93, n = 9 trials).
