# Supplementary material for: Seasonal variation in exploitative competition between honeybees and bumblebees
Source: Oecologia. 2019 Dec 16;192(2):351–61. doi: 10.1007/s00442-019-04576-w (PMC7002462; doi:10.1007/s00442-019-04576-w)
Supplement: Supplementary file 2 — Supplementary material 2 (PDF 60 kb) [file 442_2019_4576_MOESM2_ESM.pdf]

Online Resource 2

Correlation between per-day nectar standing crop volume and honey bee count on the bumble bee excluded (BBE) patch

Statistical analysis: We determined the association between nectar standing crop volume (μL) and i) daily mean honey bee count and ii) daily mean honey bee count per 1000 flowers on the BBE patch using Spearman's rank order correlation coefficient. For this we used non-pooled data from days 2 and 3 for each trial, except day 2 of Trial 1 which was removed from the analysis due to missing nectar data. In both cases daily mean honey bee count was an average of counts made between 11:00-15:00 in order to be ecologically relevant to the time at which nectar readings were taken (between 12:00-14:00).

Results: A Spearman's rank order correlation test showed a significant negative correlation between per-day mean nectar standing crop volume (μL) and i) mean honey bee count from 11:00-15:00 ($r_s = -0.672$, $P < 0.001$, n = 19 trial days) and ii) honey bee count from 11:00-15:00 per 1000 flowers ($r_s = -0.486$, $P = 0.020$, n = 19 trial days) on the BBE patch.
