# Supplementary material for: Seasonal variation in exploitative competition between honeybees and bumblebees
Source: Oecologia. 2019 Dec 16;192(2):351–61. doi: 10.1007/s00442-019-04576-w (PMC7002462; doi:10.1007/s00442-019-04576-w)
Supplement: Supplementary file 3 — Supplementary material 3 (PDF 98 kb) [file 442_2019_4576_MOESM3_ESM.pdf]

Online Resource 3

Table ESM1. Mean per-trial nectar standing crop volume (μL) and concentration (% sugar) extracted from flowers in lavender patches from which bumble bees have been excluded (BBE), honey bees have been excluded (HBE), and unmanipulated control patches (CON), across ten bee-exclusion trials in early summer (^{a}), late summer (^{b}) and autumn (^{c}) from May to September 2017. Standing crop nectar data for each trial are averaged over trial days 2 and 3 (n = 20 flowers), except Trial 1 in which nectar was extracted only on day 2 (n = 10 flowers). Concentration data are from extracted standing crop samples that were large enough such that it was possible to measure concentration using a refractometer; number of samples is shown as [n]. Kruskal Wallis χ^2 and P value for significant difference in nectar volume between patches per trial are given with Bonferroni adjustment of P values. *Post hoc* Dunn's test results for pairwise comparison of standing crop volumes between patch treatments per trial are also calculated with Bonferroni adjustment of P values. Asterisk * denotes significance at $P < 0.05$.

Trial	Patch treatment	Mean nectar standing crop (µL)	Mean nectar standing crop concentration (% sugar) [n flowers]	Per-trial Kruskal Wallis test for difference in standing crop volume between patch treatments; $\chi^2_{(DF)}$ and P value	Per-trial *post hoc* Dunn's test for pairwise comparison of standing crop volume between patch treatments; Z and (P) values
1[a]	BBE	0.1781 ± 0.0354	33.5 ± 0.5 [3]	$2.34_{(2)}$, P = 0.311	BBE – CON: 1.49 (P = 0.203)
1[a]	HBE	0.1031 ± 0.0133	33.0 ± 2.8 [2]		BBE – HBE: 0.46 (P = 0.969)
1[a]	CON	0.0758 ± 0.0097	31.8 ± 2.2 [4]		CON - HBE: -1.03 (P = 0.452)
2[a]	BBE	0.4258 ± 0.0457	36.6 ± 4.6 [19]	$40.44_{(2)}$, P < 0.001*	BBE - CON: 5.76 (P < 0.001)*
2[a]	HBE	0.0326 ± 0.0074	30.4 ±2.6 [4]		BBE - HBE: 5.21 (P < 0.001)*
2[a]	CON	0.0270 ± 0.0085	32.5 ± 0.7 [2]		CON - HBE: -0.56 (P = 0.870)
3[a]	BBE	0.5063 ± 0.0724	41.4 ± 6.7 [17]	$18.30_{(2)}$, P < 0.001*	BBE - CON: 3.23 (P = 0.002)*
3[a]	HBE	0.1602 ± 0.0496	33.7 ± 4.5[8]		BBE - HBE: 4.04 (P < 0.001)*
3[a]	CON	0.1820 ± 0.0328	32.2 ± 5.5 [9]		CON - HBE: 0.81 (P = 0.630)
4[b]	BBE	0.1018 ± 0.0331	42.8 ± 10.3 (2)	$2.98_{(2)}$, P = 0.225	BBE - CON: 0.85 (P = 0.594)
4[b]	HBE	0.0426 ± 0.0114	41.2 ± 1.6 [3]		BBE - HBE: 1.73 (P = 0.127)
4[b]	CON	0.0678 ± 0.0186	40.5 ± na [1]		CON - HBE: 0.88(P = 0.571)
5[b]	BBE	0.0658 ± 0.0117	na	$11.36_{(2)}$, P < 0.001*	BBE - CON: 3.37 (P = 0.001)*
5[b]	HBE	0.0326 ± 0.0082	29.0 ± na [1]		BBE - HBE: 1.71 (P = 0.129)
5[b]	CON	0.0201 ± 0.0064	21.0 ± na [1]		CON - HBE: -1.66 (P = 0.146)
6[b]	BBE	0.0543 ± 0.0172	30.3 ± 2.5 [2]	$11.37_{(2)}$, P < 0.001*	BBE - CON: 2.86 (P = 0.006)*
6[b]	HBE	0.0090 ± 0.0030	na		BBE - HBE: 3.51 (P < 0.001)*
6[b]	CON	0.0113 ± 0.0043	na		CON - HBE: 0.65 (P = 0.771)
7[b]	BBE	0.0367 ± 0.0085	na	$8.78_{(2)}$, P = 0.012*	BBE - CON: 2.92 (P = 0.005)*
7[b]	HBE	0.0188 ± 0.0081	na		BBE - HBE: 1.91 (P = 0.084)
7[b]	CON	0.0059 ± 0.0018	na		CON - HBE: -1.01 (P = 0.472)
8[b]	BBE	0.0707 ± 0.0128	21.5 ± 4.2 [4]	$16.12_{(2)}$, P < 0.001*	BBE - CON: 3.80 (P < 0.001)*
8[b]	HBE	0.0184 ± 0.0055	na		BBE - HBE: 3.02 (P = 0.004)*
8[b]	CON	0.0133 ± 0.0049	24.0 ± na [1]		CON - HBE: -0.79 (P = 0.648)
9[c]	BBE	0.4035 ± 0.0978	32.8 ± 5.9 [17]	$40.60_{(2)}$, P < 0.001*	BBE - CON: 5.02 (P < 0.001)*
9[c]	HBE	0.0148 ± 0.0043	na		BBE - HBE: 5.42 (P < 0.001)*
9[c]	CON	0.0184 ± 0.0048	28.5 ± 2.1 [2]		CON - HBE: 0.39 (P = 1.000)
10[c]	BBE	0.2379 ± 0.0557	27.9 ± 6.1 [14]	$21.49_{(2)}$, P < 0.001*	BBE - CON: 4.34 (P < 0.001)*
10[c]	HBE	0.0315 ± 0.0107	27.7 ± 3.8 [3]		BBE - HBE: 3.98 (P < 0.001)*
10[c]	CON	0.0166 ± 0.0036	28.8 ± 3.9 [4]		CON - HBE: -0.36 (P = 1.000)
