# Supplementary material for: Seasonal variation in exploitative competition between honeybees and bumblebees
Source: Oecologia. 2019 Dec 16;192(2):351–61. doi: 10.1007/s00442-019-04576-w (PMC7002462; doi:10.1007/s00442-019-04576-w)

Figure S1. Bumblebee (*Bombus* spp.) abundance and species composition on the control (CON) patch in ten trials from May – September 2017. Each bar represents the total number of bumblebee visits per species over trial days 2 and 3 of each trial (n = 36 = 2 days x 18 counts per day from 09:00-17:30). Species names are shown in the key. *Bombus terrestris* and *Bombus lucorum* are grouped due to the difficulty of separating these species in the field, see Methods.

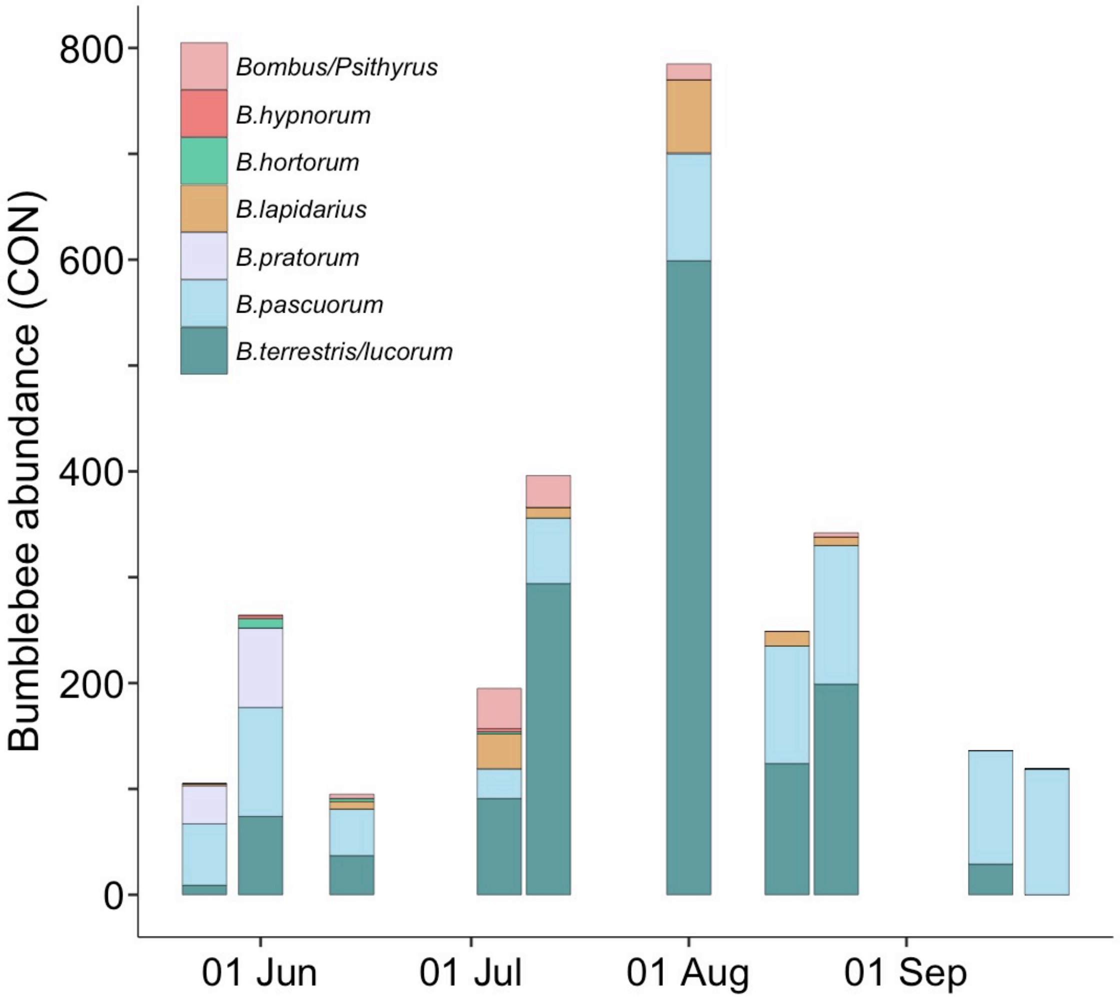

Supplement: Supplementary file 4 — Supplementary material 4 (PDF 227 kb) [file 442_2019_4576_MOESM4_ESM.pdf]
